# Convergence analysis of modulus-based matrix splitting iterative methods for implicit complementarity problems

**DOI:** 10.1186/s13660-017-1593-7

**Published:** 2018-01-03

**Authors:** An Wang, Yang Cao, Quan Shi

**Affiliations:** 1 School of Science, Nantong University, Nantong, 226019 China; 2 School of Transportation, Nantong University, Nantong, 226019 China

**Keywords:** 65F10, implicit complementarity problem, matrix splitting, modulus-based iterative method, convergence

## Abstract

In this paper, we demonstrate a complete version of the convergence theory of the modulus-based matrix splitting iteration methods for solving a class of implicit complementarity problems proposed by Hong and Li (Numer. Linear Algebra Appl. 23:629-641, 2016). New convergence conditions are presented when the system matrix is a positive-definite matrix and an $H_{+}$-matrix, respectively.

## Introduction

Consider the following implicit complementarity problem [2], abbreviated ICP, of finding a solution $u\in\mathbb{R}^{n}$ to

1.1 $$ u-m(u)\geq0,\qquad w:=Au+q\geq0,\qquad \bigl(u-m(u)\bigr)^{T}w=0, $$

where $A=(a_{ij})\in\mathbb{R}^{n\times n}$, $q=(q_{1}, q_{2}, \ldots , q_{n})^{T}\in\mathbb{R}^{n}$, and $m(\cdot)$ stands for a point-to-point mapping from $\mathbb{R}^{n}$ into itself. We further assume that $u-m(u)$ is invertible. Here $(\cdot)^{T}$ denotes the transpose of the corresponding vector. In the fields of scientific computing and economic applications, many problems can result in the solution of the ICP (1.1); see [3, 4]. In [2], the authors have shown how all kinds of complementarity problems can be transformed into the ICP (1.1). In the same paper, the authors have studied sufficient conditions of the existence and uniqueness of solution to the ICP (1.1). In particular, if the point-to-point mapping *m* is a zero mapping, then the ICP (1.1) is equivalent to

1.2 $$ u\geq0,\quad w:=Au+q\geq0,\qquad u^{T}w=0, $$

which is known as the linear complementarity problem (abbreviated LCP) [5].

In the past few decades, much more attention has been paid to find efficient iterative methods for solving the ICP (1.1). Based on a certain implicitly defined mapping *F* and the idea of iterative methods for solving LCP (1.2), Pang proposed a basic iterative method

$$u^{(k+1)}=F\bigl(u^{(k)}\bigr), \quad k\geq0, $$

where $u^{(0)}$ is a given initial vector, and established the convergence theory. For more discussions on the mapping *F* and its role in the study of the ICP (1.1), see [6]. By changing variables, Noor equivalently reformulated the ICP (1.1) as a fixed-point problem, which can be solved by some unified and general iteration methods [7]. Under some suitable conditions, Zhan et al. [8] proposed a Schwarz method for solving the ICP (1.1). By reformulating the ICP (1.1) into an optimization problem, Yuan and Yin [9] proposed some variants of the Newton method.

Recently, the modulus-based iteration methods [10], which were first proposed for solving the LCP (1.2), have attracted attention of many researchers due to their promising performance and elegant mathematical properties. The basic idea of the modulus iteration method is transforming the LCP into an implicit fixed-point equation (i.e., the absolute equation [11]). To accelerate the convergence rate of the modulus iteration method, Dong and Jiang [12] introduced a parameter and proposed a modified modulus iteration method. They showed that the modified modulus iteration method is convergent unconditionally for solving the LCP when the system matrix *A* is positive-definite. Bai [13] presented a class of modulus-based matrix splitting (MMS) iteration methods, which inherit the merits of the modulus iteration method. Some general cases of the MMS methods have been studied in [14–18]. Hong and Li extended the MMS methods to solve the ICP (1.1). Numerical results showed that the MMS iteration methods are more efficient than the well-known Newton method and the classical projection fixed-point iteration methods [1]. In this paper, we further consider the iteration scheme of the MMS iteration method and will demonstrate a complete version about the convergence theory of the MMS iteration methods. New convergence conditions are presented when the system matrix is a positive-definite matrix and an $H_{+}$-matrix, respectively.

The outline of this paper is as follows. In Section 2, we give some preliminaries. In Section 3, we introduce the MMS iteration methods for solving the ICP (1.1). We give a complete version of convergence analysis of the MMS iteration methods in Section 4. Finally, we end this paper with some conclusions in Section 5.

## Preliminaries

In this section, we recall some useful notations, definitions, and lemmas, which will be used in analyzing the convergence of the MMS iteration method for solving the ICP (1.1).

Let $A=(a_{ij}), B=(b_{ij})\in\mathbb{R}^{m\times n}$ ($1\leq i\leq m$, $1\leq j\leq n$) be two matrices. If their elements satisfy $a_{ij}\geq b_{ij}$ $(a_{ij}>b_{ij})$, then we say that $A\geq B$ $(A>B)$. If $a_{ij}\geq0$ $(a_{ij}>0)$, then $A=(a_{ij})\in\mathbb {R}^{m\times n}$ is said to be a nonnegative (positive) matrix. If $a_{ij}\leq0$ for any $i\neq j$, then *A* is called a *Z*-matrix. Furthermore, if *A* is a *Z*-matrix and $A^{-1}\geq0$, then *A* is called an *M*-matrix. A matrix $\langle A\rangle=(\langle a\rangle _{ij})\in\mathbb{R}^{n\times n}$ is called the comparison matrix of a matrix *A* if the elements $\langle a\rangle_{ij}$ satisfy

$$\langle a\rangle_{ij}= \textstyle\begin{cases} \vert a_{ij} \vert & \text{for } i=j,\\ - \vert a_{ij} \vert & \text{for } i\neq j, \end{cases}\displaystyle \quad i,j=1,2,\dots,n. $$

A matrix *A* is called an *H*-matrix if its comparison matrix $\langle A\rangle$ is an *M*-matrix, and an $H_{+}$-matrix if it is an *H*-matrix and its diagonal entries are positive; see [19]. A matrix *A* is called a symmetric positive-definite if *A* is symmetric and satisfies $x^{T}Ax>0$ for all $x\in\mathbb{R}^{n}\setminus\{0\} $. In addition, $A=F-G$ is said to be a splitting of the matrix *A* if *F* is a nonsingular matrix, and an *H*-compatible splitting if it satisfies $\langle A\rangle=\langle F\rangle- \vert G \vert $.

We use $\vert A \vert =( \vert a_{ij} \vert )$ and $\Vert A \Vert _{2}$ to denote the absolute and Euclidean norms of a matrix *A*, respectively. These symbols are easily generalized to the vectors in $\mathbb{R}^{n}$; $\sigma(A)$, $\rho (A)$, and $\operatorname{diag}(A)$ represent the spectrum, spectral radius, and diagonal part of a matrix *A*, respectively.

### Lemma 2.1

([20])

*Suppose that* $A\in\mathbb{R}^{n\times n}$ *is an* *M*-*matrix and* $B\in \mathbb{R}^{n\times n}$ *is a* *Z*-*matrix*. *Then* *B* *is called an* *M*-*matrix if* $A\leq B$.

### Lemma 2.2

([21])

*If* $A\in\mathbb{R}^{n\times n}$ *is an* $H_{+}$-*matrix*, *then* $\vert A \vert \leq \langle A\rangle^{-1}$.

### Lemma 2.3

([22])

*Let* $A\in\mathbb{R}^{n\times n}$. *Then* $\rho(A)<1$ *iff* $\lim _{n\longrightarrow\infty}A^{n}=0$.

## Modulus-based matrix splitting iteration methods for ICP

Suppose that $u-m(u)=\frac{1}{\gamma}( \vert x \vert +x)$, $w=\frac{1}{\gamma }\Omega( \vert x \vert -x)$, and $g(u)=u-m(u)$. By the assumptions we have $u=g^{-1}[{\frac{1}{\gamma}( \vert x \vert +x)}]$. To present the MMS iteration method, we first give a lemma that shows that the ICP (1.1) is equivalent to a fixed-point equation.

### Lemma 3.1

([1])

*Let* $A=F-G$ *be a splitting of the matrix* $A\in\mathbb{R}^{n\times n}$, *let* *γ* *be a positive constant*, *and let* Ω *be a positive diagonal matrix*. *For the ICP* (1.1), *the following statements hold*:

- *If* $(u, w)$ *is a solution of the ICP* (1.1), *then* $x=\frac{\gamma}{2}(u-\Omega^{-1}w-m(u))$ *satisfies the implicit fixed*-*point equation*
  3.1 $$ (\Omega+F)x=Gx+(\Omega-A) \vert x \vert -\gamma A m \biggl[g^{-1}\biggl(\frac{1}{\gamma }\bigl( \vert x \vert +x\bigr) \biggr)\biggr]-\gamma q. $$
- *If* *x* *satisfies the implicit fixed*-*point equation* (3.1), *then*
  3.2 $$ u=\frac{1}{\gamma}\bigl( \vert x \vert +x\bigr)+m(u)\quad \textit{and} \quad w=\frac {1}{\gamma}\Omega\bigl( \vert x \vert -x\bigr) $$
  *is a solution of the ICP* (1.1).

Define

$$V=\bigl\{ v: v-m(v)\geq0, Av+q\geq0\bigr\} . $$

Then based on the implicit fixed-point equation (3.1), Hong and Li [1] established the following MMS iteration methods for solving the ICP (1.1).

### Method 3.1

([1] The MMS iteration method for ICP)

Step 1: *Given* $\epsilon> 0$, $u^{(0)}\in V$, *set* $k:=0$.
Step 2: *Find the solution* $u^{(k+1)}$:
  1. *Calculate the initial vector*
    $$\begin{aligned} x^{(0)}=\frac{\gamma}{2}\bigl(u^{(k)}-\Omega^{-1} w^{(k)}-m\bigl(u^{(k)}\bigr)\bigr), \end{aligned}$$
    *set j*:=0.
  2. *Iteratively compute* $x^{(j+1)}\in\mathbb{R}^{n}$ *by solving the equations*
    $$\begin{aligned} (\Omega+F)x^{(j+1)}=Gx^{(j)}+(\Omega-A) \bigl\vert x^{(j)} \bigr\vert -\gamma A m\bigl(u^{(k)}\bigr)-\gamma q, \end{aligned}$$
  3. $$\begin{aligned} u^{(k+1)}=\frac{1}{\gamma}\bigl( \bigl\vert x^{(j+1)} \bigr\vert +x^{(j+1)}\bigr)+m\bigl(u^{(k)}\bigr). \end{aligned}$$
Step 3: *If* $\mathrm{RES}= \vert (Au^{(k+1)}+q)^{T}(u^{(k+1)}-m(u^{(k+1)})) \vert <\epsilon$, *then stop*; *otherwise*, *set* $k:=k+1$ *and return to step* 2.

Method 3.1 converges to the unique solution of ICP (1.1) under mild conditions and has a faster convergence rate than the classical projection fixed-point iteration methods and the Newton method [1]. However, Method 3.1 cannot be directly applied to solve the ICP (1.1). On one hand, the authors did not specify how to solve $w^{(k)}$. On the other hand, step 2(2) is actually an inner iteration at the *k*th outer iteration. The outer iteration information should be presented in the MMS iteration method. To better show how the MMS iteration method works, we give a complete version as follows.

### Method 3.2

(The MMS iteration method for ICP)

Step 1: *Given* $\epsilon> 0$, $u^{(0)}\in V$, *set* $k:=0$.
Step 2: *Find the solution* $u^{(k+1)}$:
  1. *Calculate the initial vector*
    3.3 $$\begin{aligned} \begin{aligned} &w^{(k)}=Au^{(k)}+q, \\ & x^{(0, k)}=\frac{\gamma}{2}\bigl(u^{(k)}- \Omega^{-1} w^{(k)}-m\bigl(u^{(k)}\bigr)\bigr), \end{aligned} \end{aligned}$$
    *set* $j:=0$.
  2. *Iteratively compute* $x^{(j+1, k)}\in\mathbb{R}^{n}$ *by solving the equations*
    3.4 $$ (\Omega+F)x^{(j+1, k)}=Gx^{(j, k)}+(\Omega-A) \bigl\vert x^{(j, k)} \bigr\vert -\gamma A m\bigl(u^{(k)}\bigr)- \gamma q. $$
  3. 3.5 $$ u^{(k+1)}=\frac{1}{\gamma}\bigl( \bigl\vert x^{(j+1, k)} \bigr\vert +x^{(j+1, k)}\bigr)+m\bigl(u^{(k)} \bigr). $$
Step 3: *If* $\mathrm{RES}= \vert (Au^{(k+1)}+q)^{T}(u^{(k+1)}-m(u^{(k+1)})) \vert <\epsilon$, *then stop*; *otherwise*, *set* $k:=k+1$ *and return to step* 2.

From Method 3.1 or Method 3.2 we can see that the MMS iteration method belongs to a class of inner-outer iteration methods. In general, the convergence rate of inner iteration has great effect on total steps of the outer iteration. However, in actual implementations the inner iterations need not communicate. Note that the number of outer iterations decreases as the number of inner iteration increases. This may lead to the reduction of the total computing time, provided that the decrement of communication time is not less than the increment of computation time for the inner iterations. So, a suitable choice of the number of inner iterations is very important and can greatly improve the computing time for solving the ICP (1.1). To efficiently implement the MMS iteration method, we can fix the number of inner iterations or choose a stopping criterion about residuals of inner iterations at each outer iteration. For the inner iteration implementation aspects of the modulus-based iteration method, we refer to [12, 23] for details.

## Convergence analysis

In this section, we establish the convergence theory for Method 3.2 when $A\in\mathbb{R}^{n\times n}$ is a positive-definite matrix and an $H_{+}$-matrix, respectively.

To this end, we first assume that there exists a nonnegative matrix $N\in\mathbb{R}^{n\times n}$ such that

$$\bigl\vert m(u)-m(v) \bigr\vert \leq N \vert u-v \vert \quad\text{for all } u ,v\in{V}. $$

Further assume that $u^{(*)}\in{V}$ and $x^{(*)}$ are the solutions of the ICP (1.1) and the implicit fixed-point equation (3.1), respectively. Then by Lemma 3.1 we have the equalities

4.1 $$\begin{aligned} u^{(*)}=\frac{1}{\gamma}\bigl( \bigl\vert x^{(*)} \bigr\vert +x^{(*)}\bigr)+m\bigl(u^{(*)} \bigr) \end{aligned}$$

and

4.2 $$\begin{aligned} (\Omega+F)x^{(*)} ={}& Gx^{(*)}+(\Omega-A) \bigl\vert x^{(*)} \bigr\vert -\gamma A m\biggl[g^{-1}\biggl( \frac{1}{\gamma }\bigl( \bigl\vert x^{(*)} \bigr\vert +x^{(*)}\bigr)\biggr)\biggr]-{\gamma}q \\ ={}& Gx^{(*)}+(\Omega-A) \bigl\vert x^{(*)} \bigr\vert - \gamma A m\bigl(u^{(*)}\bigr)-{\gamma}q. \end{aligned}$$

In addition, from Method 3.2 we have

4.3 $$\begin{aligned} x^{(*)}=\frac{\gamma}{2}\bigl(u^{(*)}- \Omega^{-1} w^{(*)}-m\bigl(u^{(*)}\bigr)\bigr), \end{aligned}$$

where $w^{(*)}=Au^{(*)}+q$.

Subtracting (4.1) from (3.5) and taking absolute values on both sides, we obtain

4.4 $$\begin{aligned} \bigl\vert u^{(k+1)}-u^{(*)} \bigr\vert &= \biggl\vert m\bigl(u^{(k)}\bigr)-m\bigl(u^{(*)}\bigr)+ \frac{1}{\gamma }\bigl( \bigl\vert x^{(j+1,k)} \bigr\vert +x^{(j+1,k)}- \bigl\vert x^{(*)} \bigr\vert -x^{(*)} \bigr) \biggr\vert \\ &\leq \bigl\vert m\bigl(u^{(k)}\bigr)-m\bigl(u^{(*)}\bigr) \bigr\vert +\frac{1}{\gamma } \bigl\vert \bigl( \bigl\vert x^{(j+1,k)} \bigr\vert - \bigl\vert x^{(*)} \bigr\vert \bigr)+ \bigl(x^{(j+1,k)}-x^{(*)}\bigr) \bigr\vert \\ &\leq N \bigl\vert u^{(k)}-u^{(*)} \bigr\vert + \frac{1}{\gamma }\bigl( \bigl\vert x^{(j+1,k)}-x^{(*)} \bigr\vert + \bigl\vert x^{(j+1,k)}-x^{(*)} \bigr\vert \bigr) \\ &= N \bigl\vert u^{(k)}-u^{(*)} \bigr\vert + \frac{2}{\gamma} \bigl\vert x^{(j+1,k)}-x^{(*)} \bigr\vert . \end{aligned}$$

Similarly, subtracting (4.2) from (3.4), we have

4.5 $$\begin{aligned} &\bigl\vert x^{(j+1, k)}-x^{(*)} \bigr\vert \\ &\quad= \bigl\vert (\Omega+F)^{-1}\bigl[G\bigl(x^{(j, k)}-x^{(*)} \bigr)+(\Omega-A) \bigl( \bigl\vert x^{(j, k)} \bigr\vert - \bigl\vert x^{(*)} \bigr\vert \bigr)-\gamma A\bigl(m\bigl(u^{(k)}\bigr)-m \bigl(u^{(*)}\bigr)\bigr)\bigr] \bigr\vert \\ &\quad= \bigl\vert (\Omega+F)^{-1}\bigl[G\bigl(x^{(j, k)}-x^{(*)} \bigr)+(\Omega-F+G) \bigl( \bigl\vert x^{(j, k)} \bigr\vert - \bigl\vert x^{(*)} \bigr\vert \bigr) \\ &\qquad{}-\gamma(F-G) \bigl(m \bigl(u^{(k)}\bigr)-m\bigl(u^{(*)}\bigr)\bigr)\bigr] \bigr\vert \\ &\quad\leq \bigl( \bigl\vert (\Omega+F)^{-1}(\Omega-F) \bigr\vert +2 \bigl\vert (\Omega+F)^{-1}G \bigr\vert \bigr)\cdot \bigl\vert x^{(j, k)}-x^{(*)} \bigr\vert \\ &\qquad{} +\gamma \bigl\vert ( \Omega+F)^{-1}F \bigr\vert \cdot \bigl(I+ \bigl\vert F^{-1}G \bigr\vert \bigr)N \bigl\vert u^{(k)}-u^{(*)} \bigr\vert \\ &\quad=\delta_{1} \bigl\vert x^{(j, k)}-x^{(*)} \bigr\vert +\gamma\delta_{2}N \bigl\vert u^{(k)}-u^{(*)} \bigr\vert , \end{aligned}$$

where $\delta_{1}= \vert (\Omega+F)^{-1}(\Omega-F) \vert +2 \vert (\Omega+F)^{-1}G \vert \text{ and } \delta_{2}= \vert (\Omega+F)^{-1}F \vert (I+ \vert F^{-1}G \vert )$. Substituting (4.5) into (4.4), we obtain

4.6 $$\begin{aligned} \bigl\vert u^{(k+1)}-u^{(*)} \bigr\vert &\leq \frac{2}{\gamma}\bigl(\delta_{1} \bigl\vert x^{(j,k)}-x^{(*)} \bigr\vert +\gamma\delta _{2}N \bigl\vert u^{(k)}-u^{(*)} \bigr\vert \bigr)+N \bigl\vert u^{(k)}-u^{(*)} \bigr\vert \\ &\leq \frac{2}{\gamma}\delta_{1}^{j+1} \bigl\vert x^{(0,k)}-x^{(*)} \bigr\vert +\bigl(2\delta _{1}^{j}\delta_{2}+\cdots+2\delta_{1} \delta_{2}+2\delta _{2}+I\bigr)N \bigl\vert u^{(k)}-u^{(*)} \bigr\vert \\ &=\frac{2}{\gamma}\delta_{1}^{j+1} \bigl\vert x^{(0,k)}-x^{(*)} \bigr\vert +\delta_{3} N \bigl\vert u^{(k)}-u^{(*)} \bigr\vert , \end{aligned}$$

where $\delta_{3}=2\sum _{i=0}^{j}\delta_{1}^{i}\delta_{2}+I$. Similarly, from (3.3) and (4.3) we have

4.7 $$\begin{aligned} \bigl\vert x^{(0, k)}-x^{(*)} \bigr\vert &= \biggl\vert \frac{\gamma}{2}\bigl(u^{(k)}-\Omega^{-1} w^{(k)}-m\bigl(u^{(k)}\bigr)\bigr)-\frac {\gamma}{2} \bigl(u^{(*)}-\Omega^{-1} w^{(*)}-m \bigl(u^{(*)}\bigr)\bigr) \biggr\vert \\ &\leq \frac{\gamma}{2}\bigl( \bigl\vert u^{(k)}-u^{(*)} \bigr\vert + \bigl\vert \Omega^{-1}A \bigr\vert \cdot \bigl\vert u^{(k)}-u^{(*)} \bigr\vert +N \bigl\vert u^{(k)}-u^{(*)} \bigr\vert \bigr) \\ &= \frac{\gamma}{2}\bigl(I+ \bigl\vert \Omega^{-1}A \bigr\vert +N \bigr) \bigl\vert u^{(k)}-u^{(*)} \bigr\vert . \end{aligned}$$

Finally, substituting (4.7) into (4.6), we get

$$\begin{aligned} \bigl\vert u^{(k+1)}-u^{(*)} \bigr\vert &\leq \delta_{1}^{j+1}\bigl(I+ \bigl\vert \Omega^{-1}A \bigr\vert +N\bigr) \bigl\vert u^{(k)}-u^{(*)} \bigr\vert + \delta _{3} N \bigl\vert u^{(k)}-u^{(*)} \bigr\vert \\ &= Z \bigl\vert u^{(k)}-u^{(*)} \bigr\vert , \end{aligned}$$

where

4.8 $$\begin{aligned} Z =\bigl(\delta_{1}^{j+1}\bigl(I+ \bigl\vert \Omega^{-1}A \bigr\vert +N\bigr)+\delta_{3} N\bigr) \bigl\vert u^{(k)}-u^{(*)} \bigr\vert . \end{aligned}$$

Therefore, if $\rho(Z)<1$, then Method 3.2 converges to the unique solution of the ICP (1.1).

We summarize our discussion in the following theorem.

### Theorem 4.1

*Suppose that* $A=F-G$ *is a splitting*, $\gamma>0$ *is a positive constant*, *and* Ω *is a positive diagonal matrix*. *Let* *Z* *be defined as in* (4.8). *If* $\rho(Z)<1$, *then the sequence* $\{ u^{(k)}\}_{k=0}^{\infty}$ *generated by Method* 3.2 *converges to the unique solution* $u^{(*)}$ *of ICP* (1.1) *for any initial vector* $u^{(0)}\in{V}$.

In Theorem 4.1 a general sufficient condition is given to guarantee the convergence of the MMS iteration method. However, this condition may be useless for practical computations. In the following two subsections, some specific conditions are given when the system matrix *A* is positive-definite and an $H_{+}$-matrix, respectively.

### The case of positive-definite matrix

#### Theorem 4.2

*Assume that* $A=F-G$ *is a splitting of a positive*-*definite matrix* *A* *with* $F\in\mathbb{R}^{n\times n}$ *being symmetric positive*-*definite*, $\Omega=\omega I\in\mathbb{R}^{n\times n}$ *with* $\omega>0$, *and* *γ* *is a positive constant*. *Denote* $\eta= \Vert (\Omega +F)^{-1}(\Omega-F) \Vert _{2}+2 \Vert (\Omega+F)^{-1}G \Vert _{2}$, $\lambda= \Vert N \Vert _{2}$, *and* $\tau= \Vert F^{-1}G \Vert _{2}$. *Suppose that* *ω* *satisfies one of the following cases*:

1. *when* $\tau^{2}\mu_{\max}<\mu_{\min}$,
  4.9 $$\begin{aligned} \tau\mu_{\max}< \omega< \sqrt{\mu_{\min} \mu_{\max}}, \end{aligned}$$
2. *when* $\tau<1$ *and* $\tau^{2}\mu_{\max}<\mu_{\min}<\tau \mu_{\max}$,
  4.10 $$\begin{aligned} \sqrt{\mu_{\min}\mu_{\max}}< \omega< \frac{(1-\tau)\mu_{\min }\mu_{\max}}{\tau\mu_{\max}-\mu_{\min}}, \end{aligned}$$
3. *when* $\tau\mu_{\max}\leq\mu_{\min}$,
  4.11 $$\begin{aligned} \omega\geq\sqrt{\mu_{\min}\mu_{\max}}. \end{aligned}$$

*If* $\lambda<\frac{1-\eta}{3-\eta}$, *then for any initial vector* $u^{(0)}\in{V}$, *the iteration sequence* $\{u^{(k)}\}_{k=0}^{\infty}$ *generated by Method* 3.2 *converges to the unique solution* $u^{(*)}$ *of the ICP* (1.1).

#### Proof

By Theorem 4.1 we just need to derive sufficient conditions for $\rho(Z)<1$. Based on the definition of *Z*, we have

4.12 $$\begin{aligned} \rho(Z)={}&\rho\bigl(\delta_{1}^{j+1}\bigl(I+ \bigl\vert \Omega^{-1}A \bigr\vert +N\bigr)+\delta_{3} N \bigr) \\ \leq{}& \bigl\Vert \delta_{1}^{j+1}\bigl(I+ \bigl\vert \Omega^{-1}A \bigr\vert +N\bigr)+\delta_{3} N \bigr\Vert _{2} \\ \leq{}&\eta^{j+1}\theta+\sigma, \end{aligned}$$

where $\eta= \Vert (\Omega+F)^{-1}(\Omega-F) \Vert _{2}+2 \Vert (\Omega+F)^{-1}G \Vert _{2}$, $\theta= \Vert I+ \vert \Omega^{-1}A \vert +N \Vert _{2}$, and $\sigma= \Vert \delta_{3}N \Vert _{2}$.

If $\Omega=\omega I\in\mathbb{R}^{n\times n}$ is a diagonal and positive-definite matrix and $F\in\mathbb{R}^{n\times n}$ is a symmetric positive-definite matrix, then it is easy to check that

$$\begin{aligned} \bigl\Vert (\Omega+F)^{-1}(\Omega-F) \bigr\Vert _{2} &= \bigl\Vert (\omega I+F)^{-1}(\omega I-F) \bigr\Vert _{2} \\ &=\max_{\mu\in\sigma(F)}\frac{ \vert \omega-\mu \vert }{\omega+\mu} \\ &=\max\biggl\{ {\frac{ \vert \omega-\mu_{\min} \vert }{\omega+\mu_{\min}}},{\frac { \vert \omega-\mu_{\max} \vert }{\omega+\mu_{\max}}}\biggr\} \\ &= \textstyle\begin{cases} \frac{\mu_{\max}-\omega}{\mu_{\max}+\omega} &\text{for } \omega\leq\sqrt{\mu_{\min}\mu_{\max}},\\ \frac{\omega-\mu_{\min}}{\omega+\mu_{\min}}& \text{for } \omega\geq\sqrt{\mu_{\min}\mu_{\max},} \end{cases}\displaystyle \end{aligned}$$

and

$$\begin{aligned} 2 \bigl\Vert (\Omega+F)^{-1}G \bigr\Vert _{2} &=2 \bigl\Vert (\omega I+F)^{-1}G \bigr\Vert _{2}\leq2 \bigl\Vert (\omega I+F)^{-1}F \bigr\Vert _{2}\cdot \bigl\Vert F^{-1}G \bigr\Vert _{2}\\ & =2\max_{\mu\in\sigma(F)} \frac{\mu\tau}{\omega+\mu} =\frac{2\mu_{\max}\tau}{\omega+\mu_{\max}}. \end{aligned}$$

Hence, we have

$$\begin{aligned} \eta& = \bigl\Vert (\Omega+F)^{-1}(\Omega-F) \bigr\Vert _{2}+2 \bigl\Vert (\Omega+F)^{-1}G \bigr\Vert _{2} \\ &\leq \textstyle\begin{cases}\frac{(1+2\tau)\mu_{\max}-\omega}{\mu_{\max }+\omega} &\text{for } \omega\leq \sqrt{\mu_{\min}\mu_{\max}},\\ \frac{\omega-\mu_{\min}}{\omega+\mu_{\min}}+\frac{2\tau\mu _{\max}}{\omega+\mu_{\max}}& \text{for } \omega\geq\sqrt {\mu_{\min}\mu_{\max}.} \end{cases}\displaystyle \end{aligned}$$

Similarly to the proof of [13, Theorem 4.2], we know that $\eta<1$ if the iteration parameter *ω* satisfies one of conditions (4.9), (4.10), and (4.11). Under those conditions, we have $\lim _{j\rightarrow\infty}\eta ^{j+1}=0$. Since *θ* is a constant, we know that, for all $\epsilon>0$, there exists an integer *J* such that, for all $j\geq J$, we have the inequality

4.13 $$ \eta^{j+1}\theta< \epsilon. $$

By the definition of $\delta_{3}$ and $0<\eta<1$, we have

$$\begin{aligned} \sigma&= \Vert \delta_{3} N \Vert _{2}= \Biggl\Vert \Biggl(2\sum_{i=0}^{j}\delta_{1}^{i} \delta _{2}+I\Biggr)N \Biggr\Vert _{2} \leq\Biggl(2 \Vert \delta_{2} \Vert _{2}\sum _{i=0}^{j}\eta^{i}+1\Biggr)\lambda\\ & \leq \biggl(\frac{2 \Vert \delta_{2} \Vert _{2}}{1-\eta}+1\biggr)\lambda. \end{aligned}$$

In addition,

$$\begin{aligned} \Vert \delta_{2} \Vert _{2}&\leq \bigl\Vert ( \Omega+F)^{-1}F \bigr\Vert _{2}\bigl(1+ \bigl\Vert F^{-1}G \bigr\Vert _{2}\bigr) = \bigl\Vert (\omega I+F)^{-1}F \bigr\Vert _{2}(1+\tau) \\ &\leq\max _{\mu\in\sigma(F)}\frac{\mu(1+\tau)}{\omega+\mu} =\frac{\mu_{\max}(1+\tau)}{\omega+\mu_{\max}}. \end{aligned}$$

It is easy to check that $\Vert \delta_{2} \Vert _{2}<1$ if $\omega>\tau\mu _{\max}$. Therefore

4.14 $$ \sigma< \biggl(\frac{2}{1-\eta}+1\biggr)\lambda. $$

By (4.12), (4.13), and (4.14) we can choose $\epsilon\ll1$ such that, for all $j\geq J$,

$$ \rho(Z)< \epsilon+\biggl(\frac{2}{1-\eta}+1\biggr)\lambda. $$

As $j\rightarrow\infty$, we have $\rho(Z)<1$, provided that $\lambda <\frac{1-\eta}{3-\eta}$. This completes the proof. □

#### Remark 4.1

Although in [1] the modulus-based iteration method was proposed based on a matrix splitting, the authors just considered the following iteration scheme in analyzing the convergence

$$(\Omega+A)x^{(j+1, k)}=(\Omega-A) \bigl\vert x^{(j, k)} \bigr\vert -\gamma A m\bigl(u^{(k)}\bigr)-{\gamma}q, $$

that is, the convergence of the modulus-based matrix splitting iteration method was not actually proved in [1]. In Theorems 4.1 and 4.2, we give a complete version of the convergence of the MMS iteration method (i.e., Method 3.2). These results generalize those in [1, Theorems 4.1 and 4.2].

### The case of $H_{+}$-matrix

In this subsection, we establish the convergence property of the MMS iteration method (i.e., Method 3.2) when $A\in\mathbb {R}^{n\times n}$ is an $H_{+}$-matrix. We obtain a new convergence result.

#### Theorem 4.3

*Let* $A\in\mathbb{R}^{n\times n}$ *be an* $H_{+}$-*matrix*, *and let* $A=F-G$ *be an* *H*-*compatible splitting of the matrix* *A*, *that is*, $\langle A\rangle=\langle F\rangle- \vert G \vert $. *Let* Ω *be a diagonal and positive*-*definite matrix*, *and let* *γ* *be a positive constant*. *Denote* $\psi_{1}=(\Omega+\langle F \rangle)^{-1}(2 \vert G \vert + \vert \Omega-F \vert )$ *and* $\psi_{2}=(\Omega+\langle F\rangle)^{-1} \vert F \vert (I+ \vert F^{-1}G \vert )$. *If*

$$\Omega\geq\frac{1}{2}\operatorname{diag}(F)\quad \textit{and} \quad\biggl( \frac{2 \Vert \psi _{2} \Vert _{2}}{1- \Vert \psi_{1} \Vert _{2}}+1\biggr)\lambda< 1, $$

*then*, *for any initial vector* $u^{(0)}\in{V}$, *the iteration sequence* $\{u^{(k)}\}_{k=0}^{\infty}$ *generated by Method *3.2 *converges to the unique solution* $u^{(*)}$ *of the ICP* (1.1).

#### Proof

Since $A=F-G$ is an *H*-compatible splitting of the matrix *A*, we have

$$\langle A\rangle\leq\langle F\rangle\leq \operatorname{diag}(F). $$

By Lemmas 2.1 and 2.2 we know that $F\in\mathbb {R}^{n\times n}$ is an $H_{+}$-matrix and

$$\bigl\vert (\Omega+F)^{-1} \bigr\vert \leq\bigl(\Omega+\langle F \rangle\bigr)^{-1}. $$

For this case, (4.5) can be somewhat modified as

$$\begin{aligned} &\bigl\vert x^{(j+1,k)}-x^{(*)} \bigr\vert \\ &\quad= \bigl\vert ( \Omega+F)^{-1}\bigl[G\bigl(x^{(j,k)}-x^{(*)}\bigr)+( \Omega -A) \bigl( \bigl\vert x^{(j,k)} \bigr\vert - \bigl\vert x^{(*)} \bigr\vert \bigr)-\gamma A\bigl(m\bigl(u^{(k)}\bigr)-m \bigl(u^{(*)}\bigr)\bigr)\bigr] \bigr\vert \\ &\quad\leq \bigl(\Omega+\langle F\rangle\bigr)^{-1}\bigl(2 \vert G \vert + \bigl\vert (\Omega -F) \bigr\vert \bigr) \bigl\vert x^{(j,k)}-x^{(*)} \bigr\vert \\ &\qquad{} +\gamma\bigl(\Omega+\langle F \rangle \bigr)^{-1} \vert F \vert \bigl(I+ \bigl\vert F^{-1}G \bigr\vert \bigr)\cdot N \bigl\vert u^{(k)}-u^{(*)} \bigr\vert \\ &\quad= \psi_{1} \bigl\vert x^{(j,k)}-x^{(*)} \bigr\vert +\gamma\psi_{2}N \bigl\vert u^{(k)}-u^{(*)} \bigr\vert . \end{aligned}$$

Similarly to analysis of Theorem 4.1 with only technical modifications, we obtain

$$\begin{aligned} \bigl\vert u^{(k+1)}-u^{(*)} \bigr\vert \leq\hat{Z} \bigl\vert u^{(k)}-u^{(*)} \bigr\vert , \end{aligned}$$

where $\hat{Z}=\psi_{1}^{j+1}(I+ \vert \Omega^{-1}A \vert +N)+\psi_{3} N$ and $\psi _{3}=2\sum _{i=0}^{j}\psi_{1}^{i}\psi_{2}+I$.

Now, we turn to study the conditions for $\rho(\hat{Z})<1$ that guarantee the convergence of the MMS iteration method. Based on the definition of *Ẑ*, we have

$$\begin{aligned} \rho(\hat{Z}) &=\rho\bigl(\psi_{1}^{j+1}\bigl(I+ \bigl\vert \Omega^{-1}A \bigr\vert +N\bigr)+\psi_{3} N\bigr) \\ &\leq \bigl\Vert \psi_{1}^{j+1}\bigl(I+ \bigl\vert \Omega^{-1}A \bigr\vert +N\bigr)+\psi_{3} N \bigr\Vert _{2} \\ &\leq \bigl\Vert \psi_{1}^{j+1} \bigr\Vert _{2}\cdot\theta+ \Biggl\Vert 2\sum_{i=0}^{j} \psi _{1}^{i}\psi_{2}+I \Biggr\Vert _{2}\lambda. \end{aligned}$$

From [13, Theorem 4.3] we know that $\rho(\psi_{1})<1$ if the parameter matrix Ω satisfies $\Omega\geq\frac {1}{2}\operatorname{diag}(F)$. By Lemma 2.3 we have $\lim _{j\rightarrow\infty}\psi_{1}^{j+1}=0$. Besides, *θ* is a positive constant. Thus, for any $\epsilon_{1}>0$ (without loss of generality, $\epsilon_{1}\ll1$), there exists an integer $j_{0}$ such that, for all $j\geq j_{0}$,

$$\bigl\Vert \psi_{1}^{j+1} \bigr\Vert _{2} \theta\leq\epsilon_{1}. $$

Therefore, for all $j\geq j_{0}$, we have

$$\begin{aligned} \rho(\hat{Z}) \leq&\epsilon_{1}+\Biggl(2 \Biggl\Vert \sum _{i=0}^{j}\psi_{1}^{i} \psi_{2} \Biggr\Vert _{2}+1\Biggr)\lambda \\ \leq& \epsilon_{1}+\bigl(2 \bigl\Vert ({I-\psi_{1}})^{-1} \psi_{2} \bigr\Vert _{2}+1\bigr)\lambda \\ \leq&\epsilon_{1}+\biggl(\frac{2 \Vert \psi_{2} \Vert _{2}}{1- \Vert \psi_{1} \Vert _{2}}+1\biggr)\lambda. \end{aligned}$$

As $j\rightarrow\infty$, we have $\rho(\hat{Z})<1$ if $(\frac{2 \Vert \psi_{2} \Vert _{2}}{1- \Vert \psi_{1} \Vert _{2}}+1)\lambda<1$. This completes the proof. □

## Conclusions

In this paper, we have studied a class of modulus-based matrix splitting (MMS) iteration methods proposed in [1] for solving implicit complementarity problem (1.1). We have modified implementation of the MMS iteration method. In addition, we have demonstrated a complete version of the convergence theory of the MMS iteration method. We have obtained new convergence results when the system matrix *A* is a positive-definite matrix and an $H_{+}$-matrix, respectively.

## References

[1] Hong J-T, Li C-L (2016). Modulus-based matrix splitting iteration methods for a class of implicit complementarity problems. Numer. Linear Algebra Appl..

[2] Pang JS, Mangasarian OL, Meyer RR, Robinson SM (1981). The Implicit Complementarity Problem. Nonlinear Programming.

[3] Ferris MC, Pang JS (1997). Engineering and economic applications of complementarity problems. SIAM Rev..

[4] Billups SC, Murty KG (2000). Complementarity problems. J. Comput. Appl. Math..

[5] Cottle RW, Pang JS, Stone RE (2009). The Linear Complementarity Problem.

[6] Pang JS (1982). On the convergence of a basic iterative method for the implicit complementarity problems. J. Optim. Theory Appl..

[7] Noor M (1988). Fixed point approach for complementarity problems. J. Optim. Theory Appl..

[8] Zhan W-P, Zhou S, Zeng J-P (2000). Iterative methods for a kind of quasi-complementarity problems. Acta Math. Appl. Sin..

[9] Yuan Q, Yin H-Y (2009). Stationary point of the minimization reformulations of implicit complementarity problems. Numer. Math. J. Chin. Univ..

[10] Schäfer U (2004). On the modulus algorithm for the linear complementarity problem. Oper. Res. Lett..

[11] Mangasarian OL, Meyer RR (2006). Absolute value equations. Linear Algebra Appl..

[12] Dong J-L, Jiang M-Q (2009). A modified modulus method for symmetric positive-definite linear complementarity problems. Numer. Linear Algebra Appl..

[13] Bai Z-Z (2010). Modulus-based matrix splitting iteration methods for linear complementarity problems. Numer. Linear Algebra Appl..

[14] Bai Z-Z, Zhang L-L (2013). Modulus-based synchronous multisplitting iteration methods for linear complementarity problems. Numer. Linear Algebra Appl..

[15] Zheng N, Yin J-F (2013). Accelerated modulus-based matrix splitting iteration methods for linear complementarity problem. Numer. Algorithms.

[16] Zheng N, Yin J-F (2014). Convergence of accelerated modulus-based matrix splitting iteration methods for linear complementarity problem with an $H_{+}$-matrix. J. Comput. Appl. Math..

[17] Zhang L-L, Zhang Y-P, Ren Z-R (2015). New convergence proofs of modulus-based synchronous multisplitting iteration methods for linear complementarity problems. Linear Algebra Appl..

[18] Dong J-L, Gao J-B, Ju F-J, Shen J-H (2016). Modulus methods for nonnegatively constrained image restoration. SIAM J. Imaging Sci..

[19] Bai Z-Z (1999). On the convergence of the multisplitting methods for linear complementarity problem. SIAM J. Matrix Anal. Appl..

[20] Frommer A, Szyld DB (1992). H-splittings and two-stage iterative methods. Numer. Math..

[21] Frommer A, Mayer G (1989). Convergence of relaxed parallel multisplitting methods. Linear Algebra Appl..

[22] Varga RS (2000). Matrix Iterative Analysis.

[23] Zhang L-L (2014). Two-stage multisplitting iteration methods using modulus-based matrix splitting as inner iteration for linear complementarity. J. Optim. Theory Appl..

